# Vitamin D in Acute Campylobacteriosis–Results From an Intervention Study Applying a Clinical *Campylobacter jejuni* Induced Enterocolitis Model

**DOI:** 10.3389/fimmu.2019.02094

**Published:** 2019-09-03

**Authors:** Soraya Mousavi, Fábia Daniela Lobo de Sá, Jörg-Dieter Schulzke, Roland Bücker, Stefan Bereswill, Markus M. Heimesaat

**Affiliations:** ^1^Institute of Microbiology, Infectious Diseases and Immunology, Charité - University Medicine Berlin, Corporate Member of Freie Universität Berlin, Humboldt-Universität zu Berlin, and Berlin Institute of Health, Berlin, Germany; ^2^Institute of Clinical Physiology, Department of Gastroenterology, Infectious Diseases and Rheumatology, Charité - Universitätsmedizin Berlin, Corporate Member of Freie Universität Berlin, Humboldt-Universität zu Berlin, and Berlin Institute of Health, Berlin, Germany

**Keywords:** vitamin D, *Campylobacter jejuni*, campylobacteriosis model, intervention study, host-pathogen interaction, acute enterocolitis, intestinal epithelial barrier function

## Abstract

Human *Campylobacter* infections are progressively rising and of high socioeconomic impact. In the present preclinical intervention study we investigated anti-pathogenic, immuno-modulatory, and intestinal epithelial barrier preserving properties of vitamin D applying an acute campylobacteriosis model. Therefore, secondary abiotic IL-10^−/−^ mice were perorally treated with synthetic 25-OH-cholecalciferol starting 4 days before peroral *Campylobacter jejuni* infection. Whereas, 25-OH-cholecalciferol application did not affect gastrointestinal pathogen loads, 25-OH-cholecalciferol treated mice suffered less frequently from diarrhea in the midst of infection as compared to placebo control mice. Moreover, 25-OH-cholecalciferol application dampened *C. jejuni* induced apoptotic cell responses in colonic epithelia and promoted cell-regenerative measures. At day 6 post-infection, 25-OH-cholecalciferol treated mice displayed lower numbers of colonic innate and adaptive immune cell populations as compared to placebo controls that were accompanied by lower intestinal concentrations of pro-inflammatory mediators including IL-6, MCP1, and IFN-γ. Remarkably, as compared to placebo application synthetic 25-OH-cholecalciferol treatment of *C. jejuni* infected mice resulted in lower cumulative translocation rates of viable pathogens from the inflamed intestines to extra-intestinal including systemic compartments such as the kidneys and spleen, respectively, which was accompanied by less compromised colonic epithelial barrier function in the 25-OH-cholecalciferol as compared to the placebo cohort. In conclusion, our preclinical intervention study provides evidence that peroral synthetic 25-OH-cholecalciferol application exerts inflammation-dampening effects during acute campylobacteriosis.

## Introduction

*Campylobacter jejuni* constitute major infectious bacterial agents of zoonotic enteric morbidities with increasing prevalences worldwide (1). Humans become infected via the food chain by consumption of raw or undercooked meat derived from contaminated livestock animals or by ingestion of *C. jejuni* containing surface water (2–4). Infected individuals present with symptoms of varying degree depending on the virulence of the acquired bacterial strain on one side and the host immune status on the other (1, 5–7). Some patients display rather mild symptoms including watery diarrhea, whereas others develop acute campylobacteriosis (8, 9). These severely compromised individuals complain about abdominal cramps, fever, and inflammatory bloody diarrhea (8, 9). During infection intestinal tissues are destroyed by innate immune responses and display profound histopathological inflammatory changes such as ulcerations, crypt abscesses, and increased numbers of innate and adaptive immune cells in the colonic mucosa and lamina propria (5, 8, 10, 11). The vast majority of human infections are usually self-limiting and treated (if at all) symptomatically. Only severely compromised patients with immuno-suppressive comorbidities, for instance, require hospitalization and receive antimicrobial treatment (6, 8, 9). In rare cases, however, post-infectious sequelae such as Guillain-Barré syndrome, Miller Fisher syndrome, Reiter's syndrome, and reactive arthritis might arise with a latency of weeks to months (8, 9, 12).

Despite the progressively increasing prevalences of human campylobacteriosis, cellular, and molecular events that are involved in disease development are not yet fully understood. However, previous clinical studies revealed that in humans acute *C. jejuni* induced disease courses and post-infectious sequelae such as Guillain-Barré syndrome are triggered by the pathogenic surface molecule lipooligosaccharide (LOS) causing hyper-activation of the innate immune system in the sialylated form (13). For quite a long time *in vivo* studies have been hampered by the scarcity of appropriate animal models. This is mainly because the gastrointestinal microbiota of mice mediates a strong colconization resistance to *C. jejuni* and mice are *per se* about 10,000-fold more resistant to LOS and lipopolysaccharide (LPS) as compared to humans (14). Our group has recently shown that secondary abiotic IL-10^−/−^ mice in which the gut microbiota had been depleted by broad-spectrum antibiotic treatment can not only be effectively colonized by the pathogen upon peroral infection, but also develop key features of acute campylobacteriosis such as wasting and bloody diarrhea within 1 week (15). One major reason for these severe immunopathological responses mounting in acute ulcerative enterocolitis is the absence of colonization resistance and the lack of interleukin-10 (IL-10) providing murine resistance to *C. jejuni* LOS (16, 17). In consequence, *C. jejuni* infected IL-10^−/−^ mice display pronounced LOS induced and Toll-like receptor-4 (TLR-4) dependent innate and adaptive immune responses that are not restricted to the intestinal tract, but can also be observed in extra-intestinal including systemic compartments (15, 18–25).

Vitamin D has primarily been known for its regulatory properties in bone metabolism due to the tight control of calcium reabsortion in the intestinal tract and in bone remodeling (26). After exposure to ultraviolet (UV) B light the steroid hormone is produced in the skin from 7-dehydroxy-cholesterol followed by hydroxylation steps in the liver and the kidneys to the biologically active forms 25-hydroxy-vitamin D and 1,25-dihydroxy-vitamin D, respectively (27). After ingestion of food or supplements, circulating 25-hydroxy-vitamin D can be utilized by many cells including immune cells and intestinal intraepithelial cells expressing the 1α-hydroxylase enzyme CYP27B, whereas 24-hydroxylase CYP24A exerts counter-regulatory properties subsequently providing local 1,25-dihydroxy-vitamin D sources in a well-balanced fashion (27).

The identification of the vitamin D receptor (VDR) on peripheral blood mononuclear cells in the 1980s first pointed to immune-related functions of vitamin D (28, 29). In fact, vitamin D has been shown to be involved in modulating both, innate and adaptive immune responses (30–33) and to exert anti-inflammatory effects (34). Furthermore, several reports underline the anti-microbial properties of vitamin D (33). For instance, vitamin D could effectively inhibit the growth of Gram-positive bacterial strains such as *Staphylococcus aureus, Streptococcus pyogenes*, and *Streptococcus mutans*, but also of Gram-negative species including *Klebsiella pneumoniae* and *Escherichia coli* (35–37). In addition, the production of antimicrobial peptides such as cathelicidin and defensins are stimulated by vitamin D (38–40). Both, immune-modulatory and antimicrobial effects might be responsible for the beneficial effects of exogenous vitamin D observed in infectious morbidities caused by *Helicobacter pylori* (41) and in respiratory tract infections (42). Moreover, vitamin D has been shown to be involved in maintenance of the intestinal epithelial barrier integrity (43).

This prompted us in our present preclinical intervention study to investigate potential pathogen-lowering, immuno-modulatory, intestinal epithelial barrier preserving and hence, disease-alleviating effects of synthetic 25-OH-cholecalciferol applying a clincial model of acute campylobacterosis.

## Materials and Methods

### Ethics Statement

All animal experiments were conducted in accordance with the European Guidelines for animal welfare (2010/63/EU) following approval by the commission for animal experiments headed by the “Landesamt für Gesundheit und Soziales” (LaGeSo, Berlin, registration numbers G0172/16 and G0247/16). Twice a day clinical conditions of mice were assessed.

### Generation of Secondary Abiotic Mice

Female and male IL-10^−/−^ mice (all in C57BL/6j background) were bred and reared under specific pathogen free (SPF) conditions in the same unit of the Forschungseinrichtungen für Experimentelle Medizin (FEM, Charité–University Medicine Berlin). Three to five mice were maintained in one cage including filter tops within an experimental semi-barrier (accessible only with lab coat, overshoes, caps, and sterile gloves) under standard conditions (22–24°C room temperature, 55 ± 15% humidity, 12 h light/12 dark cycle) and had free access to autoclaved standard chow (food pellets: ssniff R/M-H, V1534-300, Sniff, Soest, Germany).

In order to assure stable gastrointestinal *C. jejuni* colonization and to override physiological colonization resistance (44), microbiota-depleted (i.e., secondary abiotic) mice were generated (44, 45). In brief, immediately post-weaning 3-week old mice were subjected to a 10-week course of broad-spectrum antibiotic treatment by adding ampicillin plus sulbactam (1 g/L; Ratiopharm, Germany), vancomycin (500 mg/L; Cell Pharm, Germany), ciprofloxacin (200 mg/L; Bayer Vital, Germany), imipenem (250 mg/L; MSD, Germany) and metronidazole (1 g/L; Fresenius, Germany) to the autoclaved drinking water (*ad libitum*) as described elsewhere (45). To assure antibiotic washout, the antibiotic cocktail was withdrawn 4 days prior infection and thus immediately before start of the vitamin D treatment.

### Vitamin D Treatment

Vitamin D treatment started 4 days before *C. jejuni* infection. Therefore, synthetic 25-OH-cholecalciferol (purchased from Sigma-Aldrich, München, Germany) was dissolved in Tween 80 (0.2% v/v) and administered to mice via the autoclaved tap water (*ad libitum*). Considering a body weight of ~25 g per mouse and a daily drinking volume of ~5 mL, the final concentration of the synthetic 25-OH-cholecalciferol solution was 2.5 μg/mL resulting in a daily treatment dosage of 500 μg per kg body weight (equivalent to 20,000 IU per kg) (46). Hence, the applied daily vitamin D dose was far beyond the toxic doses defined for rodents (i.e., 42 mg/kg/day) (47, 48) and humans (i.e., 150 mg/kg/day) (49). Age and sex matched placebo (PLC) control mice received vehicle (i.e., Tween 80) via the drinking water (*ad libitum*).

### *C. jejuni* Infection, Gastrointestinal Colonization, and Translocation

For infection, a stock solution of *C. jejuni* 81-176 strain that had been stored at −80°C was thawed, aliquots streaked onto karmali agar (Oxoid, Wesel, Germany) and incubated in a microaerophilic atmosphere at 37°C for 48 h. Immediately before peroral infection of mice, bacteria were harvested in sterile PBS (Oxoid) to a final inoculum of 10^9^ bacterial cells. Mice (3 months of age) were perorally infected on two consecutive days (i.e., days 0 and 1). Animals were continuously maintained in a sterile environment (autoclaved food and drinking water) and handled under strict aseptic conditions to prevent from contaminations.

In order to assess gastrointestinal colonization and translocation, *C. jejuni* were quantitatively assessed in fecal samples over time post-infection (p.i.) and furthermore, in luminal samples derived from distinct parts of the gastrointestinal tract (i.e., from the stomach, duodenum, ileum, and colon) and in organ homogenates at day 6 p.i. by culture as stated elsewhere (44, 50). The detection limit of viable pathogens was ≈100 CFU per g (CFU/g). To assess *C. jejuni* bacteremia, thioglycollate enrichment broths (BD Bioscience, Germany) were inoculated with ~200 μL cardiac blood of individual mice, incubated for 7 days at 37°C, and streaked onto respective media for further identification as described (44).

### Clinical Conditions

Before and after *C. jejuni* infection the clinical conditions of mice were assessed on a daily basis by using a standardized cumulative clinical score (maximum 12 points), addressing the clinical aspect/wasting (0: normal; 1: ruffled fur; 2: less locomotion; 3: isolation; 4: severely compromised locomotion, pre-final aspect), the abundance of blood in feces (0: no blood; 2: microscopic detection of blood by the Guajac method using Haemoccult, Beckman Coulter/PCD, Germany; 4: macroscopic blood visible), and diarrhea (0: formed feces; 2: pasty feces; 4: liquid feces) as described earlier (19).

### Sampling Procedures

At day 6 p.i., mice were sacrificed by isofluran inhalation (Abbott, Germany). Luminal gastrointestinal samples (from stomach, duodenum, ileum, and colon) and *ex vivo* biopsies from colon, ileum, mesenteric lymph nodes (MLN), spleen, liver, kidneys, and lungs were taken under sterile conditions. For serum cytokine measurements cardiac blood was taken. Colonic and extra-intestinal samples were collected from each mouse in parallel for microbiological, immunohistopathological, and immunological analyses. The absolute colonic and small intestinal lengths were measured with a ruler (in cm).

### Immunohistochemistry

*In situ* immunohistochemical analyses were performed in colonic *ex vivo* biopsies that had been immediately fixed in 5% formalin and embedded in paraffin as described earlier (51–54). In brief, in order to detect apoptotic epithelial cells, proliferation epithelial cells, macrophages/monocytes, T lymphocytes, and regulatory T cells (Tregs), 5 μm thin paraffin sections of *ex vivo* biopsies were stained with primary antibodies directed against cleaved caspase 3 (Asp175, Cell Signaling, Beverly, MA, USA, 1:200), Ki67 (TEC3, Dako, Denmark, 1:100), F4/80 (# 14-4801, clone BM8, eBioscience, San Diego, CA, USA, 1:50), CD3 (#N1580, Dako, 1:10), and FOXP3 (clone FJK-165, #14-5773, eBioscience, 1:100), respectively. Positively stained cells were then examined by light microscopy (magnification 100× and 400×), and for each mouse the average number of respective positively stained cells was determined within at least six high power fields (HPF, 0.287 mm^2^, 400× magnification) by a blinded independent investigator.

### Inflammatory Mediator Detection in Supernatants of Intestinal and Extra-Intestinal *ex vivo* Biopsies

Colonic *ex vivo* biopsies were cut longitudinally, washed in phosphate buffered saline (PBS; Gibco, Life Technologies, UK), and strips of ~1 cm^2^ tissue and *ex vivo* biopsies derived from MLN (3–4 lymph nodes), liver, and spleen (one half) were placed in 24-flat-bottom well-culture plates (Nunc, Germany) containing 500 μL serum-free RPMI 1640 medium (Gibco, life technologies, UK) supplemented with penicillin (100 U/mL) and streptomycin (100 μg/mL; PAA Laboratories, Germany). After 18 h at 37°C, respective culture supernatants as well as serum samples were tested for IL-6, monocyte chemoattractant protein 1 (MCP-1), tumor necrosis factor (TNF), and interferon-γ (IFN-γ) by the Mouse Inflammation Cytometric Bead Assay (CBA; BD Biosciences, Germany) on a BD FACSCanto II flow cytometer (BD Biosciences). Systemic pro-inflammatory cytokine concentrations were measured in serum samples.

### Electrophysiological Measurements

Distal colonic *ex vivo* biopsies were mounted unstripped in Ussing chambers (0.049 cm^2^ area). Transmural electrical resistance (Rt) was recorded under voltage clamp conditions by an automatic clamp device (CVC6, Fiebig Hard and Software, Berlin, Germany) at 37°C over 1 h. The bathing solution was composed of NaCl (113.6 mmol/L), NaHCO_3_ (21.0 mmol/L), KCl (5.4 mmol/L), Na_2_HPO_4_ (2.4 mmol/L), MgCl_2_ (1.2 mmol/L), CaCl_2_ (1.2 mmol/L), NaH_2_PO_4_ (0.6 mmol/L), D(+)-glucose (10.0 mmol/L), D(+)-mannose (10.0 mmol/L), beta-hydroxybutyric acid (0.5 mmol/L), and L-glutamine (2.5 mmol/L) equilibrated with carbogen gas (pH 7.4).

### Statistical Analysis

Medians and levels of significance were determined using Mann-Whitney test (GraphPad Prism v7, USA) for pairwise comparisons of not normally distributed data, and using the one-sided ANOVA with Tukey post-correction or the Kruskal-Wallis test with Dunn's post-correction for multiple comparisons as indicated. Two-sided probability (*p*) values ≤ 0.05 were considered significant. Experiments were performed in a blinded fashion and reproduced three times.

## Results

### Intestinal Pathogen Loads Over Time Following Vitamin D Treatment of *C. jejuni* Infected Mice With Acute Enterocolitis

**Figure 1:**
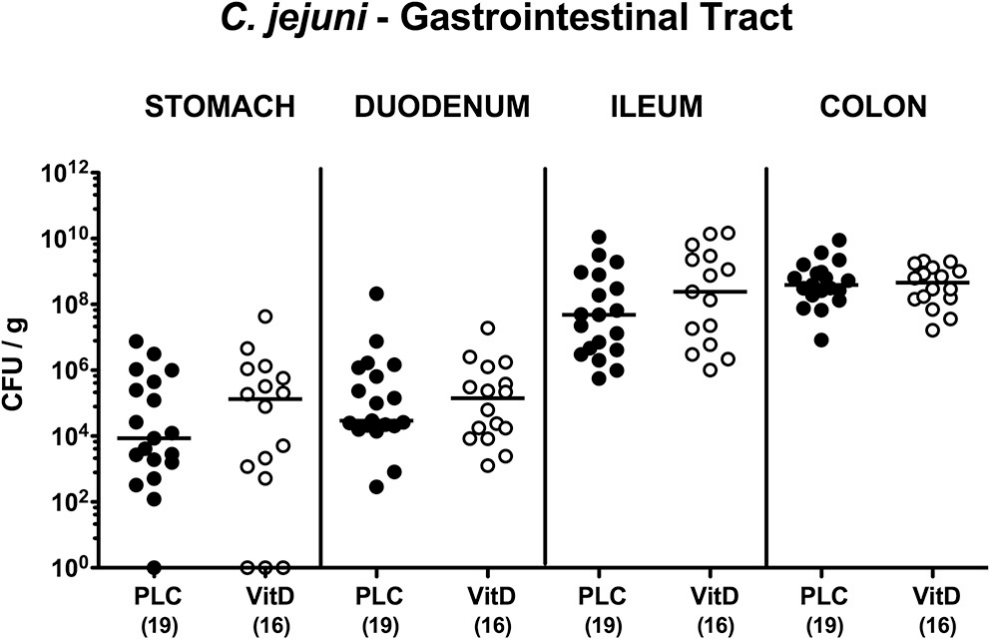
Gastrointestinal *C. jejuni* loads following vitamin D treatment of infected mice. Secondary abiotic IL-10^−/−^ mice were treated with synthetic 25-OH-cholecalciferol (vitamin D, VitD, open circles) or placebo (PLC, closed circles) via the drinking water starting 4 days before peroral *C. jejuni* 81-176 strain infection on days 0 and 1. At necropsy (i.e., day 6 post-infection), luminal *C. jejuni* loads were quantitatively assessed from each mouse in distinct gastrointestinal compartments as indicated by culture and expressed in colony forming units per g (CFU/g). Medians (black bars) and numbers of analyzed animals (in parentheses) are indicated. Data were pooled from four independent experiments.

Secondary abiotic IL-10^−/−^ mice were subjected to synthetic 25-OH-cholecalciferol treatment via the drinking water starting 4 days before *C. jejuni* infection. On two consecutive days, namely days 0 and 1, mice were then perorally challenged with 10^9^ viable pathogens by gavage. Daily cultural analyses of fecal samples revealed that 25-OH-cholecalciferol application did not affect pathogenic intestinal colonization properties as indicated by stable median fecal *C. jejuni* loads of 10^9^ CFU/g over time p.i. that did not differ between both cohorts at respective time points (n.s.; Figure S1). Upon necropsy, luminal gastrointestinal *C. jejuni* densities did not differ between 25-OH-cholecalciferol and placebo treated mice as determined in stomach, duodenum, ileum and colon at day 6 post-infection (n.s.; Figure 1). Hence, synthetic 25-OH-cholecalciferol treatment did not affect gastrointestinal *C. jejuni* loads.

### Comprehensive Survey of Clinical Conditions Over Time Following Vitamin D Treatment of *C. jejuni* Infected Mice With Acute Enterocolitis

**Figure 2:**
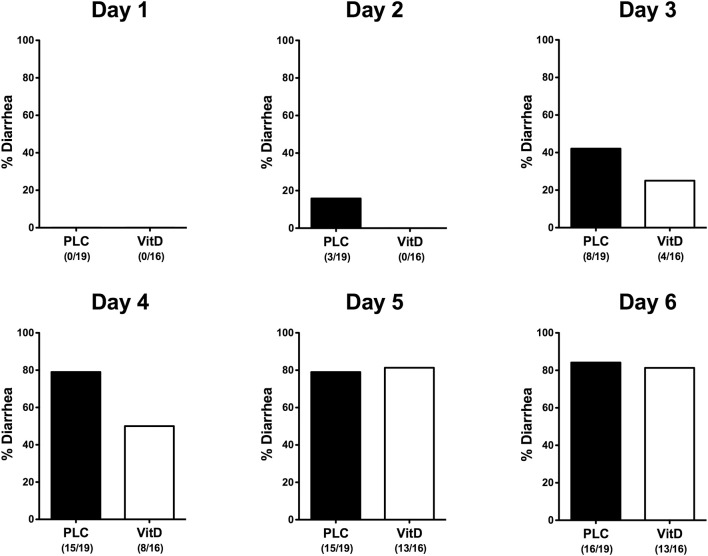
Diarrhea frequencies over time following vitamin D treatment of *C. jejuni* infected mice. Secondary abiotic IL-10^−/−^ mice were treated with synthetic 25-OH-cholecalciferol (vitamin D, VitD, white bars) or placebo (PLC, black bars) via the drinking water starting 4 days before peroral *C. jejuni* 81-176 strain infection on days 0 and 1. Occurrence of diarrhea was assessed in each mouse from day 0 until day 6 post-infection as indicated applying a standardized clinical scoring system (see Materials and Methods). Bars indicate the cumulative frequencies of diarrhea (in %). Numbers of diarrheal mice out of the total number of analyzed animals are given in parentheses. Data were pooled from four independent experiments.

Within 6 days following *C. jejuni* infection mice from either cohort developed comparably severe symptoms of acute enterocolitis as daily quantitated applying a standardized cumulative clinical scoring system (Figure S2) assessing wasting symptoms, abundance of fecal blood, and the severity of diarrhea. Whereas overall pathogen-induced clinical symptoms were comparable between the two cohorts over time (n.s.; Figure S2), cumulate relative frequencies of diarrhea were lower in 25-OH-cholecalciferol treated mice as compared to placebo controls as early as 24 h following the latest infection (i.e., day 2 p.i.) until 4 p.i. (Figure 2). Hence, synthetic 25-OH-cholecalciferol treatment results in less frequent *C. jejuni* induced diarrhea in the midst of infection.

### Macroscopic and Microscopic Inflammatory Sequelae Following Vitamin D Treatment of *C. jejuni* Infected Mice With Acute Enterocolitis

Given that intestinal inflammation is association with a significant shortening of the affected part of the intestinal tract (15, 45), we measured the lengths of both, the small and large intestines upon necropsy. In fact, *C. jejuni* infection was accompanied with shorter colons of placebo as well as of 25-OH-cholecalciferol treated mice (*p* < 0.001; Figure S3A), whereas the small intestinal lengths were virtually unaffected at day 6 p.i. (n.s.; Figure S3B). Hence, synthetic 25-OH-cholecalciferol treatment does not ameliorate *C. jejuni* induced macroscopic disease.

**Figure 3:**
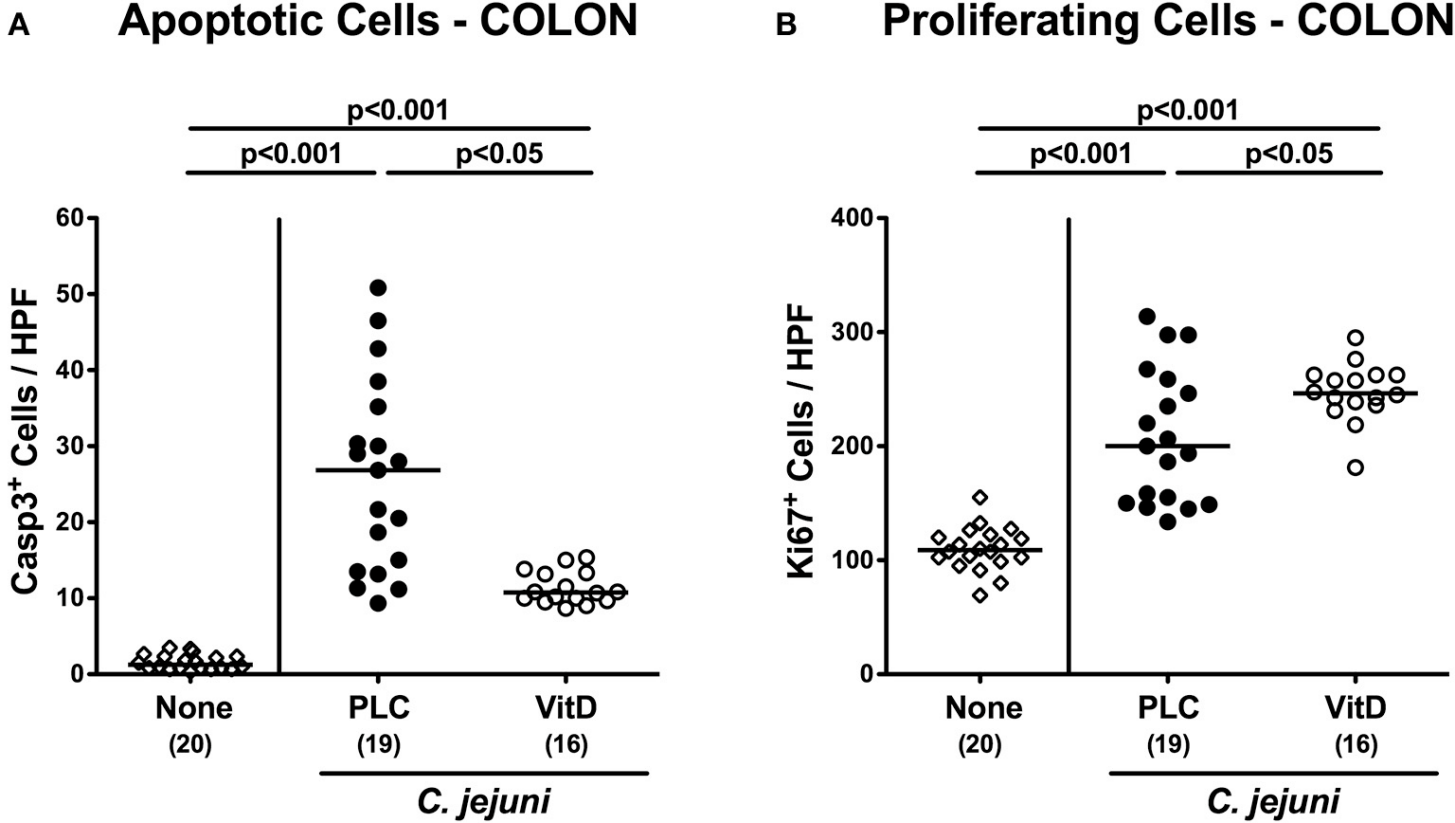
Colonic epithelial cell apoptosis and cell proliferation/regeneration following vitamin D treatment of *C. jejuni* infected mice. Secondary abiotic IL-10^−/−^ mice were treated with synthetic 25-OH-cholecalciferol (vitamin D, VitD, open circles) or placebo (PLC, closed circles) via the drinking water starting 4 days before peroral *C. jejuni* 81-176 strain infection on days 0 and 1. At necropsy (i.e., day 6 post-infection), the average numbers of colonic epithelial **(A)** apoptotic (Casp3^+^) and **(B)** proliferating (Ki67^+^) cells were assessed microscopically from six high power fields (HPF, 400× magnification) per animal in immunohistochemically stained colonic paraffin sections. Uninfected and untreated mice (none, open diamonds) served as negative control animals. Medians (black bars), levels of significance (*p*-values) assessed by the Kruskal-Wallis test and Dunn's post-correction or the one-sided ANOVA test with Tukey post-correction and numbers of analyzed animals (in parentheses) are indicated. Data were pooled from four independent experiments.

Since apoptosis is regarded a reliable parameter for the grading of intestinal inflammation (44), we further quantitatively assessed caspase3^+^ apoptotic epithelial cells in large intestinal *ex vivo* biopsies applying *in situ* immunohistochemistry. At day 6 p.i., *C. jejuni* infected mice exhibited multifold increased numbers of apoptotic cells in their colonic epithelia (*p* < 0.001), that were, however, more than 60% lower in 25-OH-cholecalciferol as compared to placebo treated mice (*p* < 0.05; Figure 3A, Figure S4A). Conversely, numbers of Ki67^+^ colonic epithelial cells indicative for cell proliferation and regeneration increased upon *C. jejuni* infection (*p* < 0.001), but more distinctly following 25-OH-cholecalciferol as compared to placebo treatment (*p* < 0.05; Figure 3B, Figure S4B). Hence, synthetic 25-OH-cholecalciferol treatment dampens *C. jejuni* induced apoptotic cell responses and promotes cell regenerative measures counteracting intestinal cell damage upon pathogenic exposure.

### Intestinal Immune Cell Responses Following Vitamin D Treatment of *C. jejuni* Infected Mice With Acute Enterocolitis

**Figure 4:**
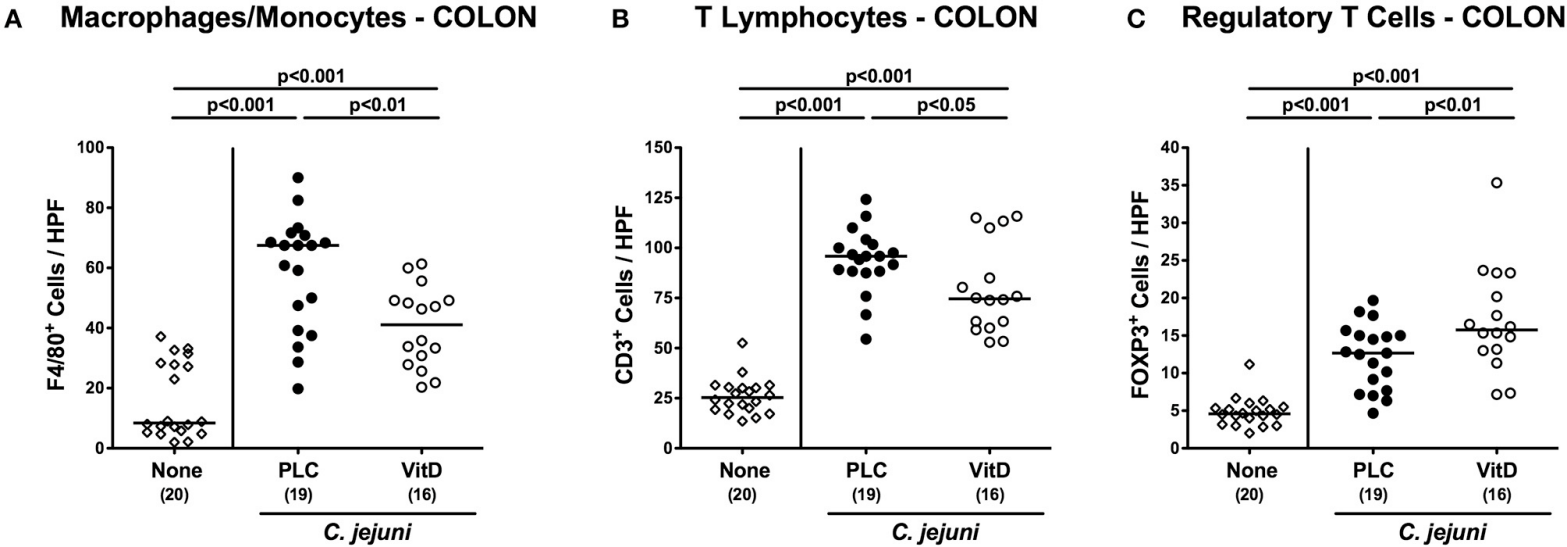
Colonic immune cell responses following vitamin D treatment of *C. jejuni* infected mice. Secondary abiotic IL-10^−/−^ mice were treated with synthetic 25-OH-cholecalciferol (vitamin D, VitD, open circles) or placebo (PLC, closed circles) via the drinking water starting 4 days before peroral *C. jejuni* 81-176 strain infection on days 0 and 1. At necropsy (i.e., day 6 post-infection), the average numbers of **(A)** macrophages and monocytes (F4/80^+^), **(B)** T lymphocytes (CD3^+^) and **(C)** regulatory T cells (FOXP3+) were assessed microscopically from six high power fields (HPF, 400× magnification) per animal in immunohistochemically stained colonic paraffin sections. Uninfected and untreated mice (none, open diamonds) served as negative control animals. Medians (black bars), levels of significance (*p*-values) assessed by the one-sided ANOVA test with Tukey post-correction and numbers of analyzed animals (in parentheses) are indicated. Data were pooled from four independent experiments.

We further quantitatively surveyed both, innate and adaptive immune cell responses in the large intestinal tract following synthetic 25-OH-cholecalciferol treatment of *C. jejuni* infected mice by immunohistochemical staining of colonic paraffin sections. As early as 6 days upon *C. jejuni* infection, numbers of F4/80^+^ innate immune cell subsets including macrophages and monocytes had increased in the large intestinal mucosa and lamina propria (*p* < 0.001), but less distinctly in 25-OH-cholecalciferol as compared to placebo challenged mice (*p* < 0.01; Figure 4A, Figure S4C). Similarly, *C. jejuni* induced increases in adaptive immune cells such as CD3^+^ lymphocytes, were less pronounced in the 25-OH-cholecalciferol vs. placebo cohort at day 6 p.i. (*p* < 0.05, VitD vs. PLC; Figure 4B, Figure S4D). Interestingly, numbers of FOXP3^+^ regulatory T cells (Treg) were slightly higher following vitamin D as compared to placebo treated *C. jejuni* infected mice (*p* < 0.01; Figure 4C, Figure S4E). Hence, synthetic 25-OH-cholecalciferol treatment results in less pronounced *C. jejuni* induced intestinal responses of distinct innate and adaptive immune cell populations.

### Intestinal Pro-inflammatory Mediator Secretion Following Vitamin D Treatment of *C. jejuni* Infected Mice With Acute Enterocolitis

**Figure 5:**
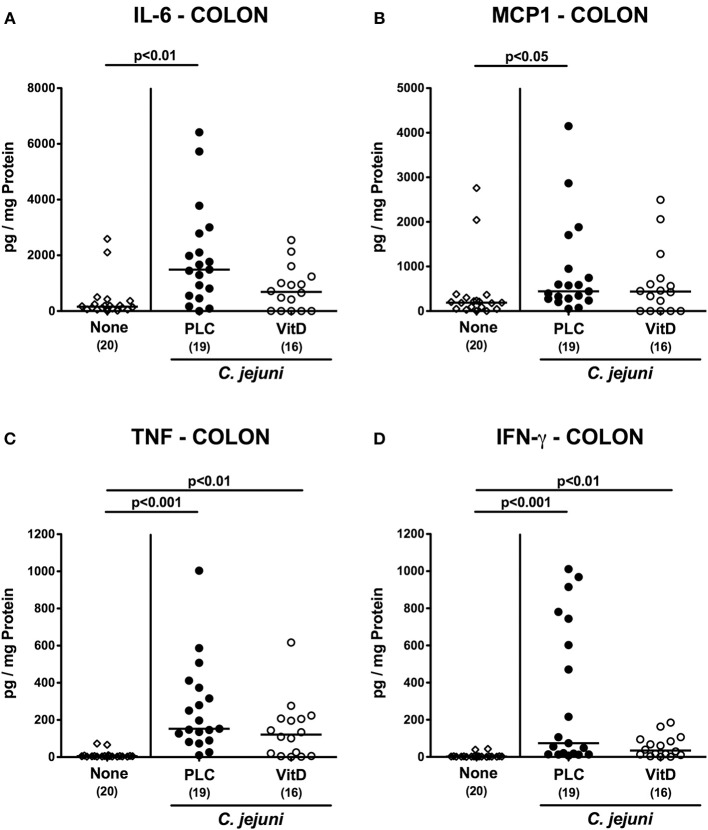
Colonic secretion of pro-inflammatory mediators following vitamin D treatment of *C. jejuni* infected mice. Secondary abiotic IL-10^−/−^ mice were treated with synthetic 25-OH-cholecalciferol (vitamin D, VitD, open circles) or placebo (PLC, closed circles) via the drinking water starting 4 days before peroral *C. jejuni* 81-176 strain infection on days 0 and 1. At necropsy (i.e., day 6 post-infection), **(A)** IL-6, **(B)** MCP1, **(C)** TNF, and **(D)** IFN-γ concentrations were determined in supernatants derived from colonic *ex vivo* biopsies. Uninfected and untreated mice (none, open diamonds) served as negative control animals. Medians (black bars), levels of significance (*p*-values) assessed by the Kruskal-Wallis test and Dunn's post-correction and numbers of analyzed animals (in parentheses) are indicated. Data were pooled from four independent experiments.

**Figure 6:**
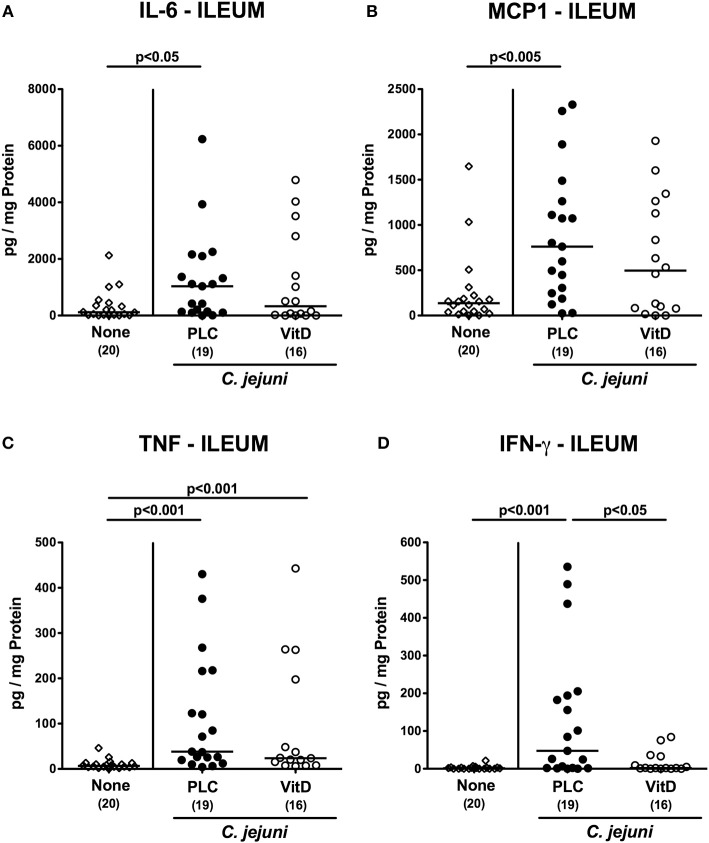
Ileal secretion of pro-inflammatory mediators following vitamin D treatment of *C. jejuni* infected mice. Secondary abiotic IL-10^−/−^ mice were treated with synthetic 25-OH-cholecalciferol (vitamin D, VitD, open circles) or placebo (PLC, closed circles) via the drinking water starting 4 days before peroral *C. jejuni* 81-176 strain infection on days 0 and 1. At necropsy (i.e., day 6 post-infection), **(A)** IL-6, **(B)** MCP1, **(C)** TNF, and **(D)** IFN-γ concentrations were determined in supernatants derived from ileal *ex vivo* biopsies. Uninfected and untreated mice (none, open diamonds) served as negative control animals. Medians (black bars), levels of significance (*p*-values) assessed by the Kruskal-Wallis test and Dunn's post-correction and numbers of analyzed animals (in parentheses) are indicated. Data were pooled from four independent experiments.

We next measured pro-inflammatory mediators in intestinal *ex vivo* biopsies. At day 6 following *C. jejuni* infection increased IL-6 and MCP-1 concentrations could be assessed in the colon of placebo (*p* < 0.01 and *p* < 0.05, respectively), but not 25-OH-cholecalciferol treated mice (Figures 5A,B). *C. jejuni* induced increases in large intestinal TNF and IFN-γ concentrations (*p* < 0.05–0.001 vs. none), however, were unaffected by 25-OH-cholecalciferol challenge (n.s. vs. PLC; Figures 5C,D). In line, ileal IL-6 and MCP-1 as well as IFN-γ levels were elevated upon *C. jejuni* infection of mice from the placebo (*p* < 0.05–0.01), but not from the 25-OH-cholecalciferol cohort (Figures 6A,B,D), whereas like in the colon, ileal TNF concentrations were comparably elevated at day 6 post-infection of either cohort (*p* < 0.001; Figure 6C). Hence, synthetic 25-OH-cholecalciferol treatment of *C. jejuni* infected mice results in less pronounced secretion of distinct pro-inflammatory mediators in the intestinal tract.

### Extra-Intestinal Inflammatory Immune Responses Following Vitamin D Treatment of *C. jejuni* Infected Mice

**Figure 7:**
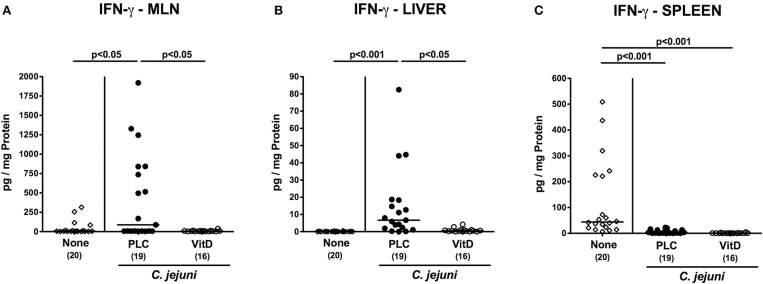
Extra-intestinal IFN-γ secretion following vitamin D treatment of *C. jejuni* infected mice. Secondary abiotic IL-10^−/−^ mice were treated with synthetic 25-OH-cholecalciferol (vitamin D, VitD, open circles) or placebo (PLC, closed circles) via the drinking water starting 4 days before peroral *C. jejuni* 81-176 strain infection on days 0 and 1. At necropsy (i.e., day 6 post-infection), IFN-γ concentrations were determined in supernatants of *ex vivo* biopsies derived from **(A)** mesenteric lymph nodes (MLN), **(B)** liver, and **(C)** spleen. Uninfected and untreated mice (none, open diamonds) served as negative control animals. Medians (black bars), levels of significance (*p*-values) assessed by the Kruskal-Wallis test and Dunn's post-correction and numbers of analyzed animals (in parentheses) are indicated. Data were pooled from four independent experiments.

We further asked whether the 25-OH-cholecalciferol mediated anti-inflammatory effects were restricted to the intestinal tract or also effective in extra-intestinal compartments. In fact, IFN-γ concentrations were lower in MLN and liver of 25-OH-cholecalciferol as compared to placebo treated mice at day 6 p.i. (*p* < 0.05; Figures 7A,B). Interestingly, *C. jejuni* infection resulted in decreased IFN-γ secretion in splenic *ex vivo* biopsies irrespective of the treatment regimen (*p* < 0.001; Figure 7C). Hence, synthetic 25-OH-cholecalciferol treatment of *C. jejuni* infected mice resulted in less distinct IFN-γ secretion in MLN and liver.

### Systemic Pro-inflammatory Mediator Secretion Following Vitamin D Treatment of *C. jejuni* Infected Mice With Acute Enterocolitis

We next addressed whether synthetic 25-OH-cholecalciferol treatment might alleviate systemic *C. jejuni* induced pro-inflammatory immune responses. At day 6 p.i., mice from either cohort exhibited comparably elevated IL-6, MCP1, TNF, and IFN-γ serum concentrations (*p* < 0.001 vs. none; Figure S5). Hence, synthetic 25-OH-cholecalciferol treatment does not affect *C. jejuni* induced systemic pro-inflammatory mediator secretion.

### Bacterial Translocation Following Vitamin D Treatment of *C. jejuni* Infected Mice With Acute Enterocolitis

**Figure 8:**
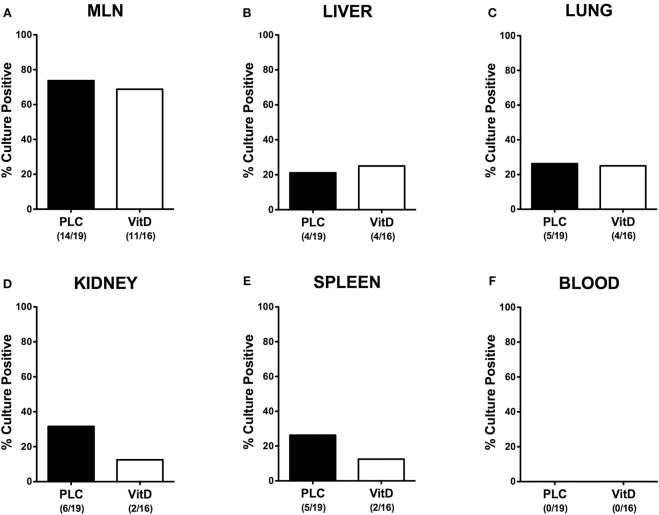
Bacterial translocation following vitamin D treatment of *C. jejuni* infected mice. Secondary abiotic IL-10^−/−^ mice were treated with synthetic 25-OH-cholecalciferol (vitamin D, VitD, white bars) or placebo (PLC, black bars) via the drinking water starting 4 days before peroral *C. jejuni* 81-176 strain infection on days 0 and 1. Upon necropsy (at day 6 post-infection), the abundance of viable pathogens was assessed in *ex vivo* biopsies taken from **(A)** mesenteric lymph nodes (MLN), **(B)** liver, **(C)** lung, **(D)** kidney, **(E)** spleen, and **(F)** cardiac blood by culture. The cumulative relative translocation rates of *C. jejuni* into the respective compartment out of four independent experiments are indicated in %. The numbers of culture-positive mice out of the total numbers of analyzed animals are given in parentheses.

We further asked whether synthetic 25-OH-cholecalciferol treatment had an impact of the translocation rates of viable pathogens from the infected intestines to extra-intestinal including systemic tissue sites. Whereas, *C. jejuni* could be cultured at similar frequencies from MLN, liver and lungs derived from 25-OH-cholecalciferol and placebo treated mice (Figures 8A–C), cumulative pathogenic translocation rates were lower in the kidneys (12.5 vs. 31.6%) and the spleen (12.5 vs. 26.3%) taken from the former as compared to the latter at day 6 p.i. (Figures 8D,E). Notably, all blood cultures remained *C. jejuni* negative (Figure 8F). Hence, synthetic 25-OH-cholecalciferol treatment was associated with lower cumulative translocation rates of *C. jejuni* originating from the inflamed intestines to the kidneys and the spleen.

### Colonic Epithelial Barrier Changes Following Vitamin D Treatment of *C. jejuni* Infected Mice With Acute Enterocolitis

**Figure 9:**
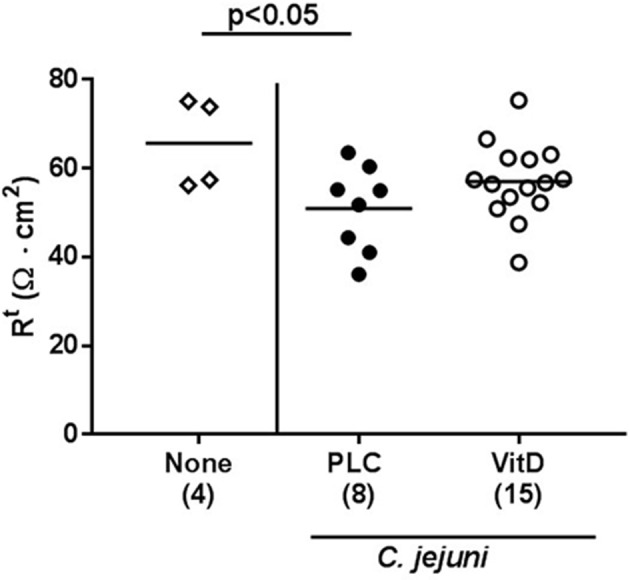
Colonic transmural electrical resistance following vitamin D treatment of *C. jejuni* infected mice. Secondary abiotic IL-10^−/−^ mice were treated with synthetic 25-OH-cholecalciferol (vitamin D, VitD, open circles) or placebo (PLC, closed circles) via the drinking water starting 4 days before peroral *C. jejuni* 81-176 strain infection on days 0 and 1. At necropsy (i.e., day 6 post-infection), the transmural electrical resistance of distal colon was measured in Ussing chambers as described in Materials and Methods. Uninfected and untreated mice (none, open diamonds) served as negative control animals. Medians (black bars), levels of significance (*p*-values) assessed by the Kruskal-Wallis test and Dunn's post-correction and numbers of analyzed animals (in parentheses) are indicated. Data were pooled from two independent experiments.

Given the lower cumulative pathogenic translocation rates we assessed whether synthetic 25-OH-cholecalciferol treatment resulted in a less compromised colonic epithelial barrier function in *C. jejuni* infected mice. Therefore, we performed electrophysiological resistance measurements of colonic *ex vivo* biopsies in the Ussing chamber. In fact, transmural resistances were lower in the large intestines derived from placebo, but not 25-OH-cholecalciferol treated mice at day 6 p.i. as compared to uninfected and untreated control animals (*p* < 0.05; Figure 9). Hence, synthetic 25-OH-cholecalciferol treatment results in uncompromised colonic epithelial barrier function following *C. jejuni* infection.

## Discussion

Due to the pleiotropic beneficial effects of vitamin D in health and disease, the application of vitamin D as safe dietary supplement is currently discussed as promising option for the adjunct treatment and prophylaxis of various immunopathological morbidities including infectious diseases, intestinal inflammatory conditions, and cancer, for instance (33, 55). In our present vitamin D intervention study applying a clinical acute campylobacteriosis model, prophylactic synthetic 25-OH-cholecalciferol application starting 4 days prior murine infection resulted in dampened *C. jejuni* induced intestinal and extra-intestinal inflammatory sequalae, but could not lower the high intestinal pathogen loads of more than 10^9^ viable *C. jejuni* per g feces. In support, recent reports revealed that the beneficial effects of vitamin D during gastrointestinal infection with distinct bacterial species such as *Salmonella* (56) or *Listeria monocytogenes* (57) are rather due to the pleiotropic immuno-modulatory than direct anti-microbial properties of the steroid hormone. In addition, one needs to take into consideration, that, in contrary to humans, the expression of the antimicrobial peptide cathelicidin in mice is not regulated by vitamin D, given that in the murine cathelicidin gene promoter the vitamin D response element is missing (58, 59). This could explain our observation that external 25-OH-cholecalciferol application, even in high doses, did not reduce intestinal *C. jejuni* burdens. However, it is tempting to speculate that this could be the case in humans.

Despite the high intestinal pathogenic burdens, 25-OH-cholecalciferol treated mice suffered less frequently from diarrhea in the midst of campylobacteriosis development as compared to placebo controls, but exhibited comparable macroscopic disease at the end of the observation period. Notably, the macroscopic outcome particularly in such a non-selflimiting detrimental intestinal infection and inflammation model is due to the sum effect of many different intestinal, extra-intestinal and systemic events within this hyper-inflammatory scenario (24). It is therefore remarkable, that less distinct *C. jejuni* induced apoptosis of colonic epithelial cells, whereas, conversely, large intestinal cell regenerative properties counteracting pathogen-induced cell damage were promoted upon 25-OH-cholecalciferol application in mice suffering from acute enterocolitis. In support, the intestinal epithelial vitamin D receptor has been shown to regulate mucosal inflammation by suppressing intestinal epithelial cell apoptosis (60). Less severe colonic apoptosis upon 25-OH-cholecalciferol treatment was accompanied by less distinct immune cell responses upon *C. jejuni* infection, which is supported by several studies showing that vitamin D regulates both, innate and adaptive immunity (61–63). In our study, lower numbers of innate immune cell populations such as macrophages and monocytes could be assessed in the colonic mucosa and lamina propria of *C. jejuni* infected mice that had been pretreated with synthetic 25-OH-cholecalciferol. In line, recent reports revealed that vitamin D stimulation of antigen presenting cells including macrophages and dendritic cells resulted in decreased pro-inflammatory mediator secretion (59, 64). In addition, colonic mucosal numbers of T lymphocytes were lower in 25-OH-cholecalciferol as compared to placebo treated mice with *C. jejuni* induced enterocolitis. In fact, T cells have been shown to be direct and indirect targets of vitamin D (65, 66). Previous *in vitro, ex vivo*, and *in vivo* studies revealed that vitamin D treatment of T cells and of mice resulted in less distinct T cell proliferation and in decreased T helper cell (Th)−1 dependent secretion of pro-inflammatory cytokines and subsequently in ameliorated inflammation (66, 67). In our present study, the colonic concentrations of pro-inflammatory mediators including IL-6 and MPC-1 measured in 25-OH-cholecalciferol pretreated, *C. jejuni* infected mice were comparable to those obtained from naive controls. In support, vitamin D was shown to reduce recruitment of innate immune cells such as monocytes and to decrease IL-6 and MCP-1 releases upon *in vitro* stimulation (68). Notably, the 25-OH-cholecalciferol associated decreased pro-inflammatry mediator secretion was not restricted to the large intestines, the major predilection site of *C. jejuni* induced enterocolitis (15, 69). In fact, *C. jejuni* induced increased secretion of IL-6, MCP-1, and additionally of IFN-γ could be observed in the terminal ileum of mice from the placebo, but not from the 25-OH-cholecalciferol treatment cohort. Interestingly, as opposed to 25-OH-cholecalciferol related decreases in large intestinal T cell numbers, higher numbers of (potentially anti-inflammatory) FOXP3^+^ regulatory T cells could be assessed in the colonic mucosa and lamina propria of 25-OH-cholecalciferol vs. placebo treated mice with enterocolitis. In support, recent studies reported that vitamin D results in enhanced recruitment of regulatory T cells to inflamed tissue sites (70–72). Given that we did not perform co-staining analyses in our present study, however, we can not answer which specific immune cell subset was expressing FOXP3.

Remarkably, the pro-inflammatory immune response-dampening effects of exogenous 25-OH-cholecalciferol were not restricted to the intestinal tract, but were also effective in extra-intestinal compartments given that *C. jejuni* induced IFN-γ secretion was less pronounced in MLN draining the inflamed intestines and in the liver upon 25-OH-cholecalciferol treatment. In line, previous studies provide evidence that vitamin D application or even skin exposure to UV light could ameliorate or prevent from liver inflammation due to vitamin D mediated dampening of immune cellular responses and inhibition of liver apoptosis, for instance (73, 74).

At the first glance unexpectedly, *C. jejuni* infection was associated with decreases in splenic IFN-γ concentrations in either cohort. One possible explanation might be that upon pathogenic infection leukocytes were recruited from the spleen to the site of infection in order to limit pathogenic spread. One could have expected an even more prominent effect following synthetic 25-OH-cholecalciferol application due to the known immune cell recruiting properties of vitamin D (75).

*C. jejuni* infection results in impaired epithelial barrier function *in vitro* (76) and campylobacteriosis is characterized by a leaky gut syndrome facilitating pathogenic translocation from the inflamed intestines to extra-intestinal including systemic compartments (15, 69). Given that vitamin D has been shown to preserve epithelial barrier function (75), we assessed potential 25-OH-cholecalciferol mediated effects on pathogenic translocation frequencies in our preclinical survey. In fact, when taking results of the four independent experiment together, *C. jejuni* could be cultured less frequently from the kidneys and the spleen of infected mice following 25-OH-cholecalciferol as compared to placebo treatment, whereas cumulative relative *C. jejuni* translocation rates to MLN, liver and lungs were comparable. Of note, all blood cultures remained *C. jejuni* negative, irrespective of the treatment regimen. One needs to take into consideration, however, that soluble bacterial molecules including LOS and others might have been transported via the circulation contributing to the observed extra-intestinal collateral damages of *C. jejuni* infection. Nevertheless, the observed inflammation-alleviating effects upon 25-OH-cholecalciferol application were further accompanied by a less compromised colonic epithelial barrier function in 25-OH-cholecalciferol as compared to placebo treated, *C. jejuni* infected mice. This in turn very likely reduced the risk of spread of both, viable bacteria and soluble bacterial molecules in the former vs. the latter. We therefore hypothesize that the 25-OH-cholecalciferol associated anti-inflammatory effects in particular prevent from further bacteria-induced damages in this acute *C. jejuni* induced inflammation model.

## Conclusion

Our preclinical intervention study provides evidence that prophylactic peroral synthetic 25-OH-cholecalciferol application dampens intestinal and extra-intestinal inflammatory responses during acute campylobacteriosis in the clinical mouse model applied here. Further studies are needed in order to define appropriate vitamin D doses for the prevention and combat of distinct gastrointestinal infectious morbidities in humans.

## Data Availability

All datasets generated for this study are included in the manuscript/Supplementary Files.

## Author Contributions

SM performed experiments, analyzed data, and co-wrote paper. FL and RB performed experiments, analyzed data, and co-edited paper. J-DS and SB provided advice in experimental design, critically discussed results, and co-edited paper. MH designed and performed experiments, analyzed data, and wrote paper.

### Conflict of Interest Statement

The authors declare that the research was conducted in the absence of any commercial or financial relationships that could be construed as a potential conflict of interest.

## Supplementary Material

Figure S1 Fecal *C. jejuni* loads over time following vitamin D treatment of infected mice. Secondary abiotic IL-10^−/−^ mice were treated with **(A)** placebo (closed circles) or **(B)** synthetic 25-OH-cholecalciferol (vitamin D, open circles) via the drinking water starting four days before peroral *C. jejuni* 81-176 strain infection on day (d) 0 and d1. Fecal *C. jejuni* loads were quantitatively assessed from each mouse on a daily basis post-infection (p.i.) by culture and expressed in colony forming units per g (CFU/g). Medians (black bars) and numbers of analyzed animals (in parentheses) are indicated. Data were pooled from four independent experiments.

Figure S2 Kinetic survey of overall clinical conditions following vitamin D treatment of *C. jejuni* infected mice. Secondary abiotic IL-10^−/−^ mice were treated with **(A)** placebo (closed circles) or **(B)** synthetic 25-OH-cholecalciferol (vitamin D, open circles) via the drinking water starting four days before peroral *C. jejuni* 81-176 strain infection on days 0 and 1. Clinical symptoms were quantitatively assessed applying a standardized clinical scoring system from d0 until d6 post-infection (see Materials and Methods). Uninfected and untreated mice (none, open diamonds) served as negative control animals. Medians (black bars) and numbers of analyzed animals (in parentheses) are indicated. Data were pooled from four independent experiments.

Figure S3 Intestinal lengths following vitamin D treatment of *C. jejuni* infected mice. Secondary abiotic IL-10^−/−^ mice were treated with synthetic 25-OH-cholecalciferol (vitamin D, VitD, open circles) or placebo (PLC, closed circles) via the drinking water starting 4 days before peroral *C. jejuni* 81-176 strain infection on days 0 and 1. At necropsy (i.e., day 6 post-infection), the absolute lengths of the **(A)** colon and **(B)** small intestines were measured with a ruler. Uninfected and untreated mice (none, open diamonds) served as negative control animals. Medians (black bars), levels of significance (*p*-values) assessed by one-sided ANOVA test with Tukey post-correction and numbers of analyzed animals (in parentheses) are indicated. Data were pooled from four independent experiments.

Figure S4 Representative photomicrographs illustrating apoptotic and proliferating epithelial as well as immune cells responses in large intestines following vitamin D treatment of *C. jejuni* infected mice. Secondary abiotic IL-10^−/−^ mice were treated with synthetic 25-OH-cholecalciferol (vitamin D) or placebo via the drinking water starting 4 days before peroral *C. jejuni* 81-176 strain infection on days 0 and 1. Naive mice served as uninfected and untreated controls. Photomicrographs reepresentative for four independent experiments illustrate the average numbers of **(A)** apoptotic epithelial cells (Casp3+), **(B)** proliferating epithelial cells, **(C)** macrophages and monocytes (F4/80+), **(D)** T lymphocytes (CD3+) and **(E)** regulatory T cell (Treg, FOXP3+) in at least six high power fields (HPF) as quantitatively assessed in ileal paraffin sections applying *in situ* immunohistochemistry at day 6 post-infection (100× magnification, scale bar 100 μm).

Figure S5 Systemic secretion of pro-inflammatory mediators following vitamin D treatment of *C. jejuni* infected mice. Secondary abiotic IL-10^−/−^ mice were treated with synthetic 25-OH-cholecalciferol (vitamin D, VitD, open circles) or placebo (PLC, closed circles) via the drinking water starting 4 days before peroral *C. jejuni* 81-176 strain infection on days 0 and 1. At necropsy (i.e., day 6 post-infection), **(A)** IL-6, **(B)** MCP1, **(C)** TNF, and **(D)** IFN-γ concentrations were determined in serum samples. Uninfected and untreated mice (none, open diamonds) served as negative control animals. Medians (black bars), levels of significance (*p*-values) assessed by the Kruskal-Wallis test and Dunn's post-correction and numbers of analyzed animals (in parentheses) are indicated. Data were pooled from four independent experiments.

## Abbreviations

CBA: Cytometric Bead Array
CFU: colony-forming units
HPF: High power fields
IFN: interferon
IL: interleukin
LOS: Lipo-oligosaccharide
LPS: Lipo-polysaccharide
MCP-1: monocyte chemoattractant protein 1
MLN: mesenteric lymph nodes
PBS: phosphate buffered saline
p.i.: post-infection
PLC: Placebo
Rt: Transmural electrical resistance
SPF: specific pathogen free
Th: T helper cell
TLR: toll-like receptor
TNF: tumor necrosis factor
Treg: regulatory T cells
UV: ultraviolet
VDR: vitamin D receptor
VitD: Vitamin D.
