# Supplementary figures and images for: Vitamin D in Acute Campylobacteriosis–Results From an Intervention Study Applying a Clinical Campylobacter jejuni Induced Enterocolitis Model
Source: Front Immunol. 2019 Sep 3;10:2094. doi: 10.3389/fimmu.2019.02094 (PMC6735268; doi:10.3389/fimmu.2019.02094)

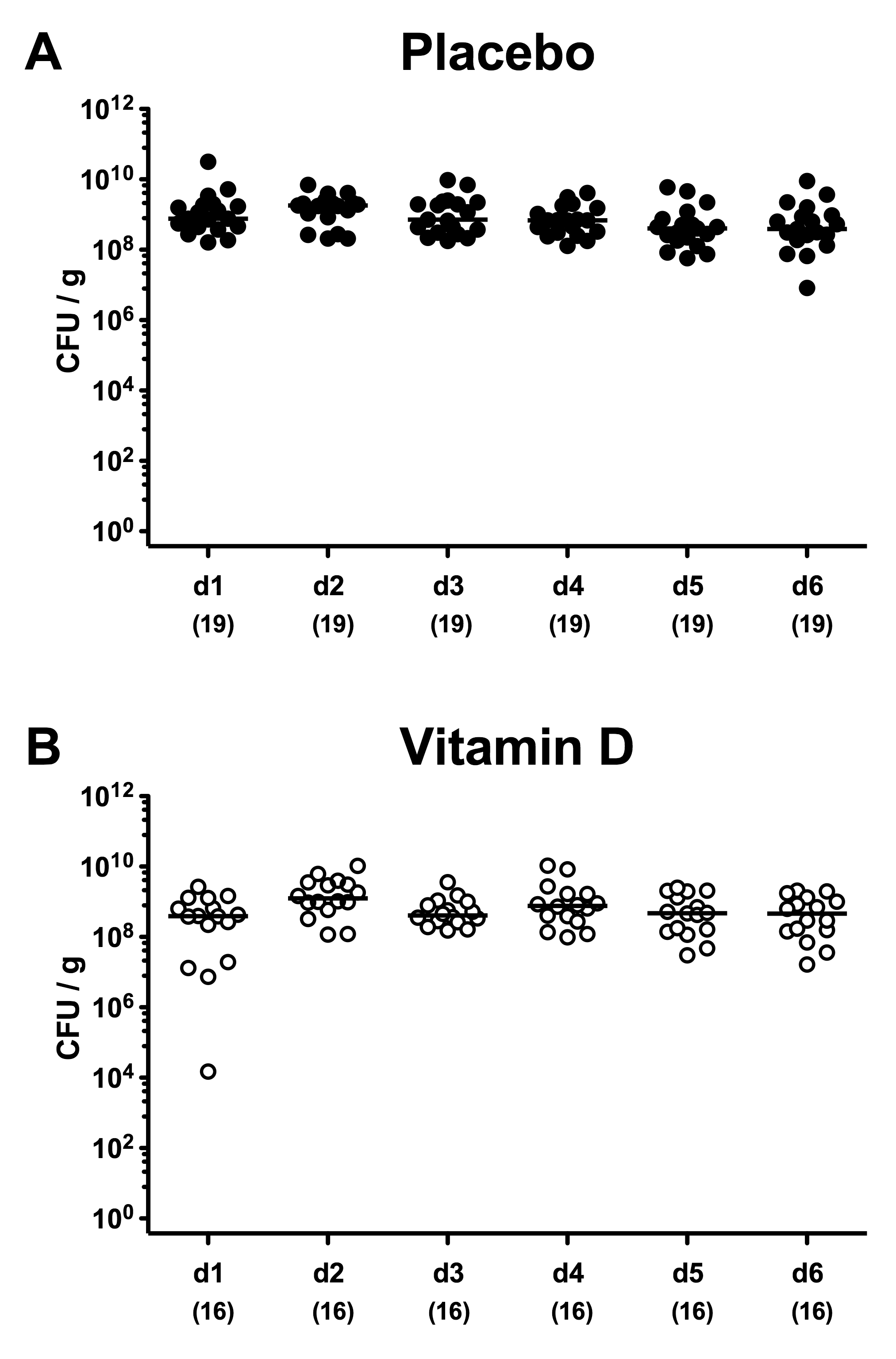

Supplement: Figure S1 — Fecal C. jejuni loads over time following vitamin D treatment of infected mice. Secondary abiotic IL-10−/− mice were treated with (A) placebo (closed circles) or (B) synthetic 25-OH-cholecalciferol (vitamin D, open circles) via the drinking water starting four days before peroral C. jejuni 81-176 strain infection on day (d) 0 and d1. Fecal C. jejuni loads were quantitatively assessed from each mouse on a daily basis post-infection (p.i.) by culture and expressed in colony forming units per g (CFU/g). Medians (black bars) and numbers of analyzed animals (in parentheses) are indicated. Data were pooled from four independent experiments. [file Image_1.TIFF]

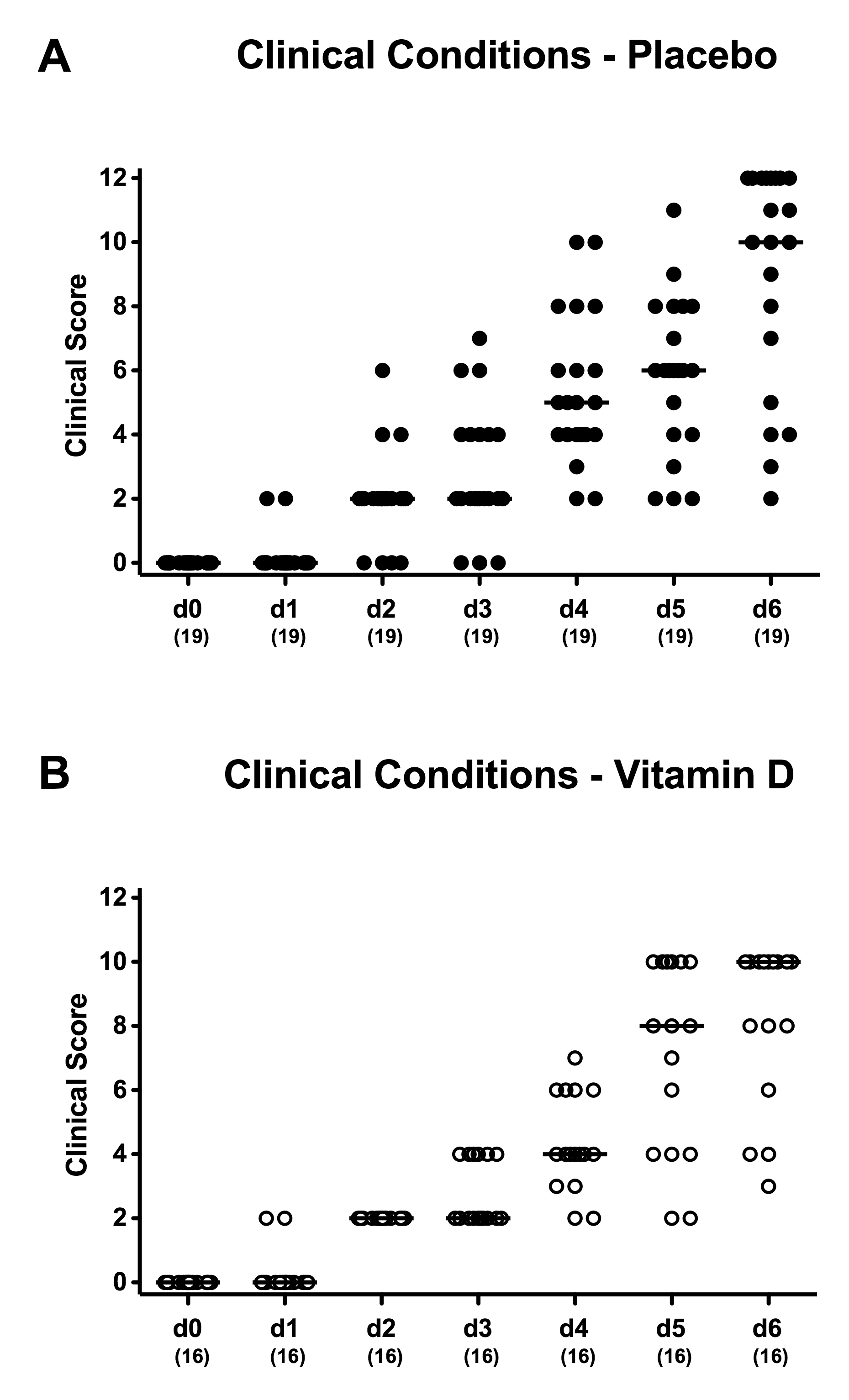

Supplement: Figure S2 — Kinetic survey of overall clinical conditions following vitamin D treatment of C. jejuni infected mice. Secondary abiotic IL-10−/− mice were treated with (A) placebo (closed circles) or (B) synthetic 25-OH-cholecalciferol (vitamin D, open circles) via the drinking water starting four days before peroral C. jejuni 81-176 strain infection on days 0 and 1. Clinical symptoms were quantitatively assessed applying a standardized clinical scoring system from d0 until d6 post-infection (see Materials and Methods). Uninfected and untreated mice (none, open diamonds) served as negative control animals. Medians (black bars) and numbers of analyzed animals (in parentheses) are indicated. Data were pooled from four independent experiments. [file Image_2.TIFF]

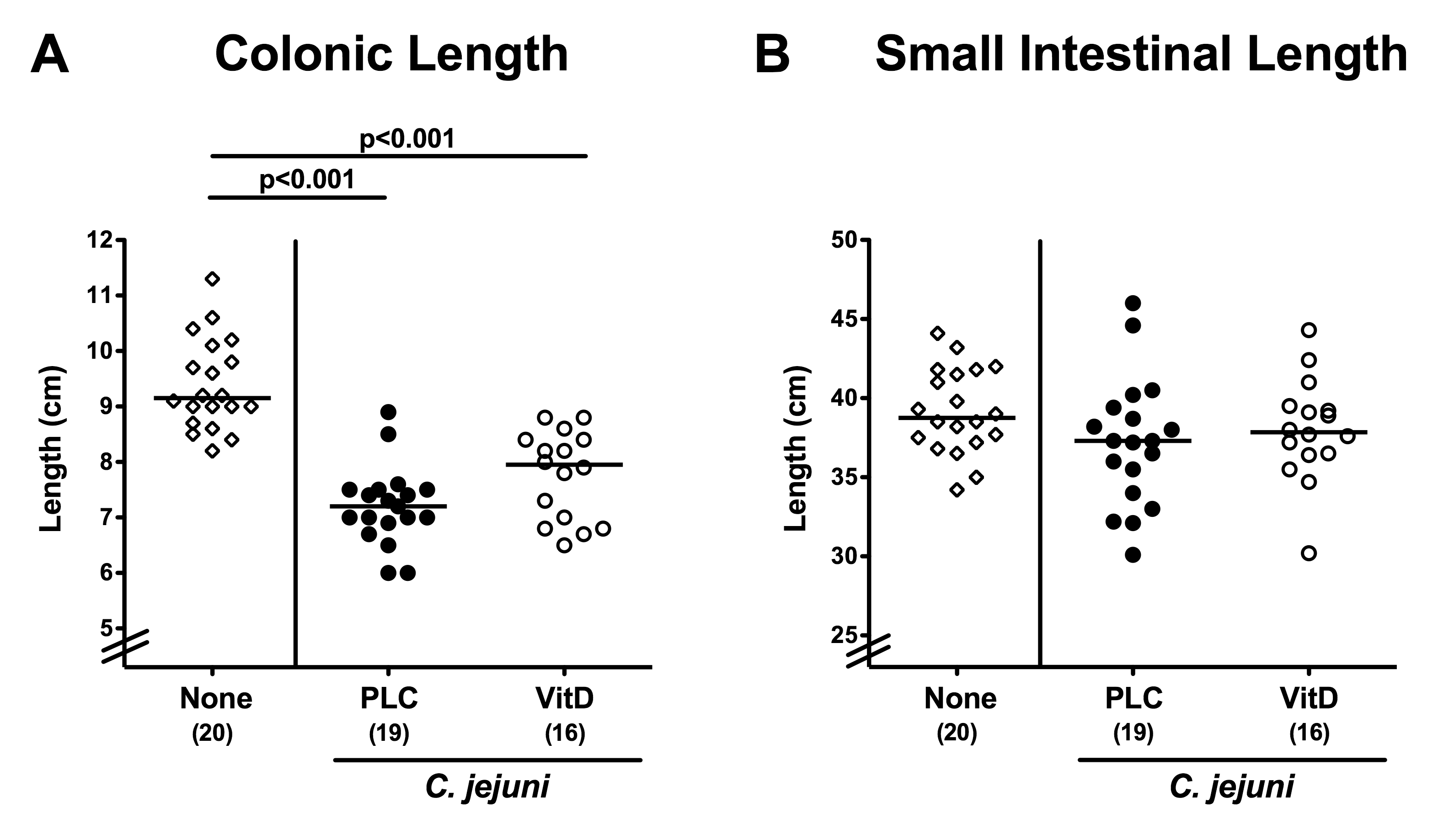

Supplement: Figure S3 — Intestinal lengths following vitamin D treatment of C. jejuni infected mice. Secondary abiotic IL-10−/− mice were treated with synthetic 25-OH-cholecalciferol (vitamin D, VitD, open circles) or placebo (PLC, closed circles) via the drinking water starting 4 days before peroral C. jejuni 81-176 strain infection on days 0 and 1. At necropsy (i.e., day 6 post-infection), the absolute lengths of the (A) colon and (B) small intestines were measured with a ruler. Uninfected and untreated mice (none, open diamonds) served as negative control animals. Medians (black bars), levels of significance (p-values) assessed by one-sided ANOVA test with Tukey post-correction and numbers of analyzed animals (in parentheses) are indicated. Data were pooled from four independent experiments. [file Image_3.TIFF]

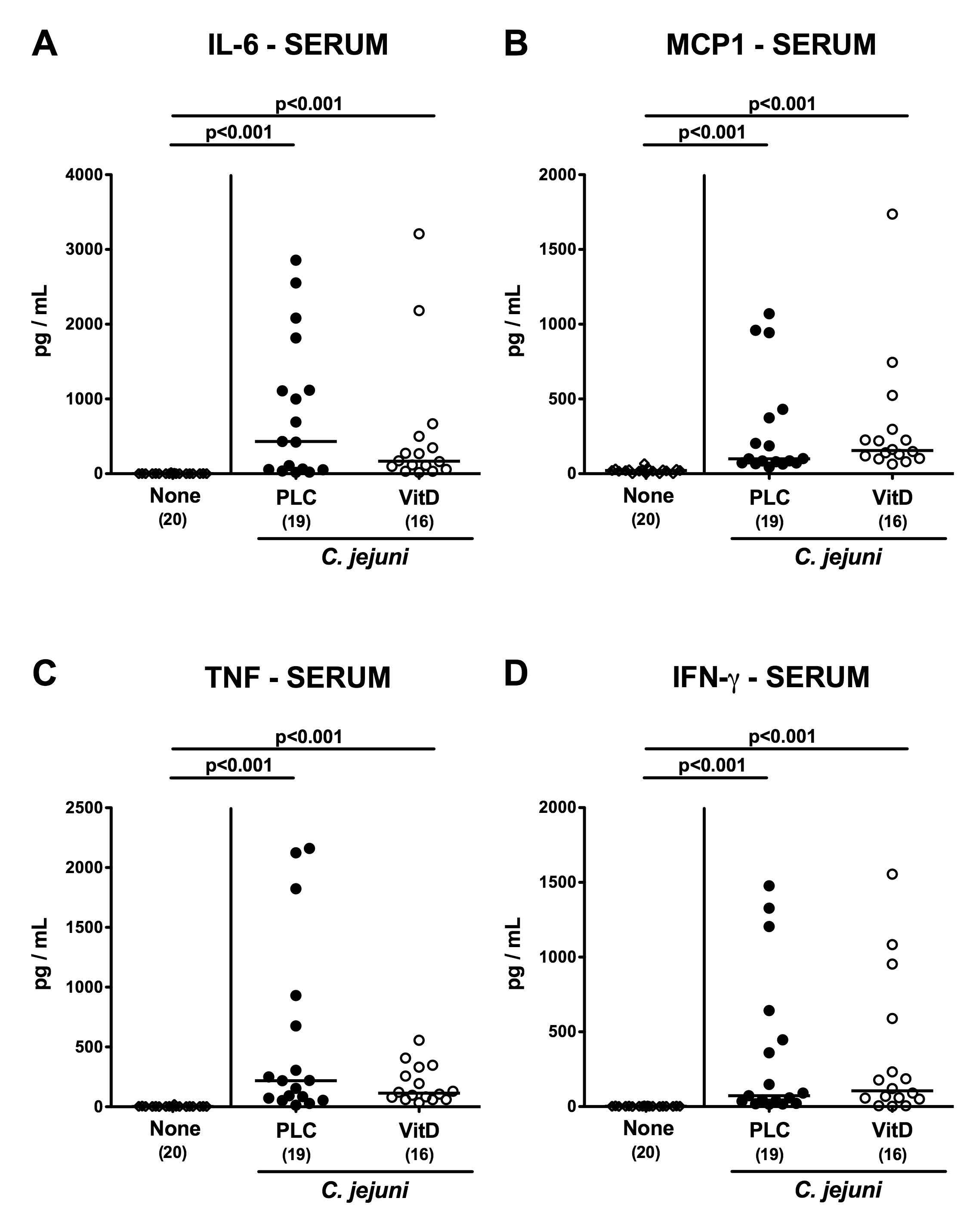

Supplement: Figure S5 — Systemic secretion of pro-inflammatory mediators following vitamin D treatment of C. jejuni infected mice. Secondary abiotic IL-10−/− mice were treated with synthetic 25-OH-cholecalciferol (vitamin D, VitD, open circles) or placebo (PLC, closed circles) via the drinking water starting 4 days before peroral C. jejuni 81-176 strain infection on days 0 and 1. At necropsy (i.e., day 6 post-infection), (A) IL-6, (B) MCP1, (C) TNF, and (D) IFN-γ concentrations were determined in serum samples. Uninfected and untreated mice (none, open diamonds) served as negative control animals. Medians (black bars), levels of significance (p-values) assessed by the Kruskal-Wallis test and Dunn's post-correction and numbers of analyzed animals (in parentheses) are indicated. Data were pooled from four independent experiments. [file Image_5.tiff]
