# Supplementary material for: Vitamin D in Acute Campylobacteriosis–Results From an Intervention Study Applying a Clinical Campylobacter jejuni Induced Enterocolitis Model
Source: Front Immunol. 2019 Sep 3;10:2094. doi: 10.3389/fimmu.2019.02094 (PMC6735268; doi:10.3389/fimmu.2019.02094)
Supplement: Figure S4 — Representative photomicrographs illustrating apoptotic and proliferating epithelial as well as immune cells responses in large intestines following vitamin D treatment of C. jejuni infected mice. Secondary abiotic IL-10−/− mice were treated with synthetic 25-OH-cholecalciferol (vitamin D) or placebo via the drinking water starting 4 days before peroral C. jejuni 81-176 strain infection on days 0 and 1. Naive mice served as uninfected and untreated controls. Photomicrographs reepresentative for four independent experiments illustrate the average numbers of (A) apoptotic epithelial cells (Casp3+), (B) proliferating epithelial cells, (C) macrophages and monocytes (F4/80+), (D) T lymphocytes (CD3+) and (E) regulatory T cell (Treg, FOXP3+) in at least six high power fields (HPF) as quantitatively assessed in ileal paraffin sections applying in situ immunohistochemistry at day 6 post-infection (100× magnification, scale bar 100 μm). [file Image_4.pdf]

A Apoptotic Cells - COLON

None **Placebo** **Vitamin D**

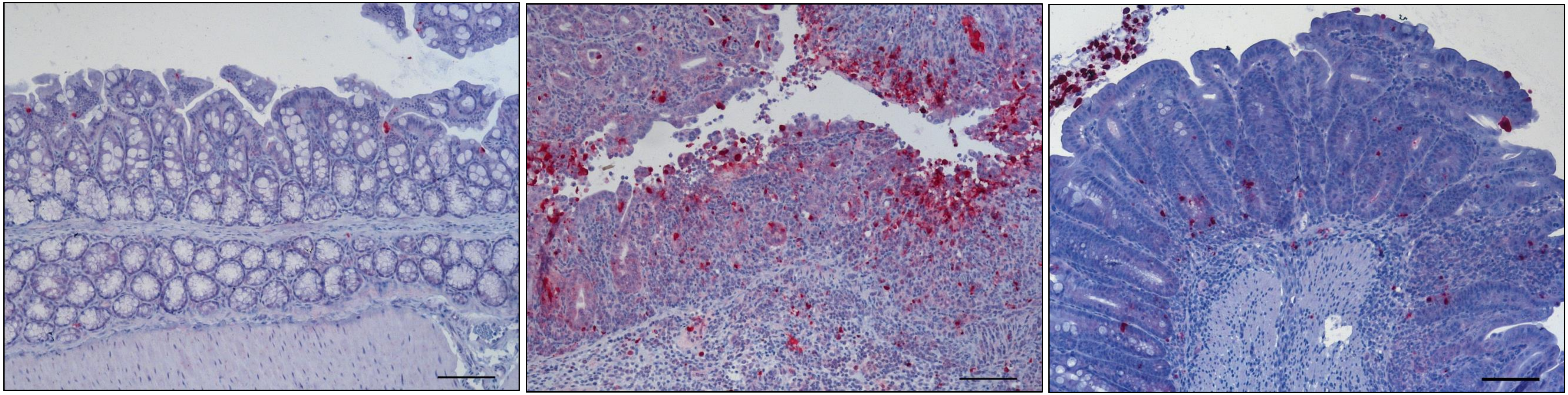

(100 x magnification, scale bar 100 μm)

B Proliferating Cells - COLON

None **Placebo** **Vitamin D**

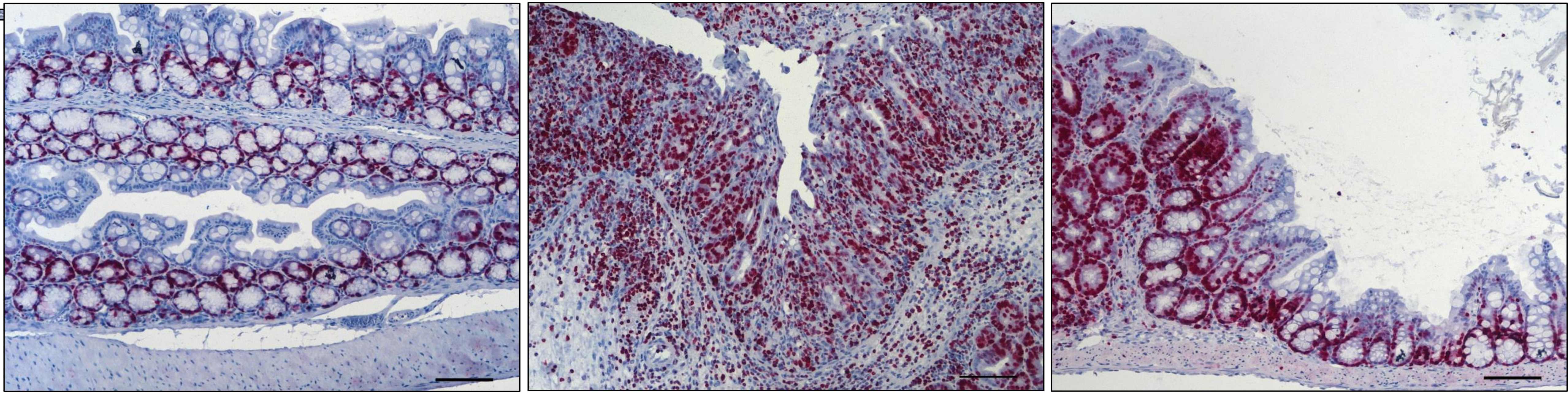

(100 x magnification, scale bar 100 μm)

C Macrophages / Monocytes - COLON

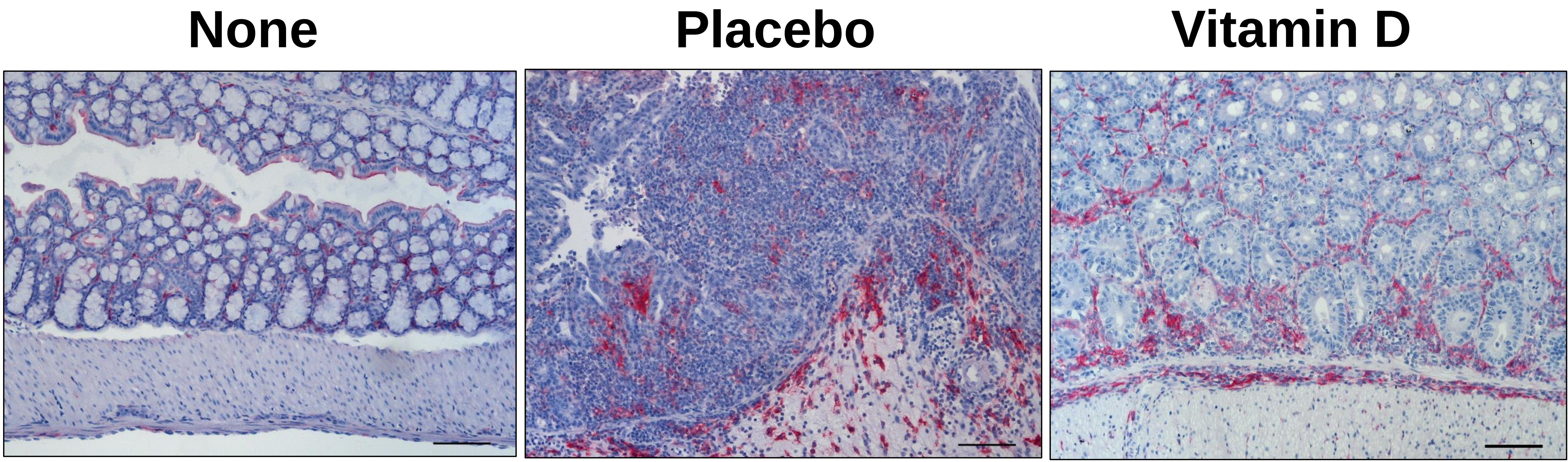

(100 x magnification, scale bar 100 µm)

D

T Lymphocytes - COLON

None **Placebo** **Vitamin D**

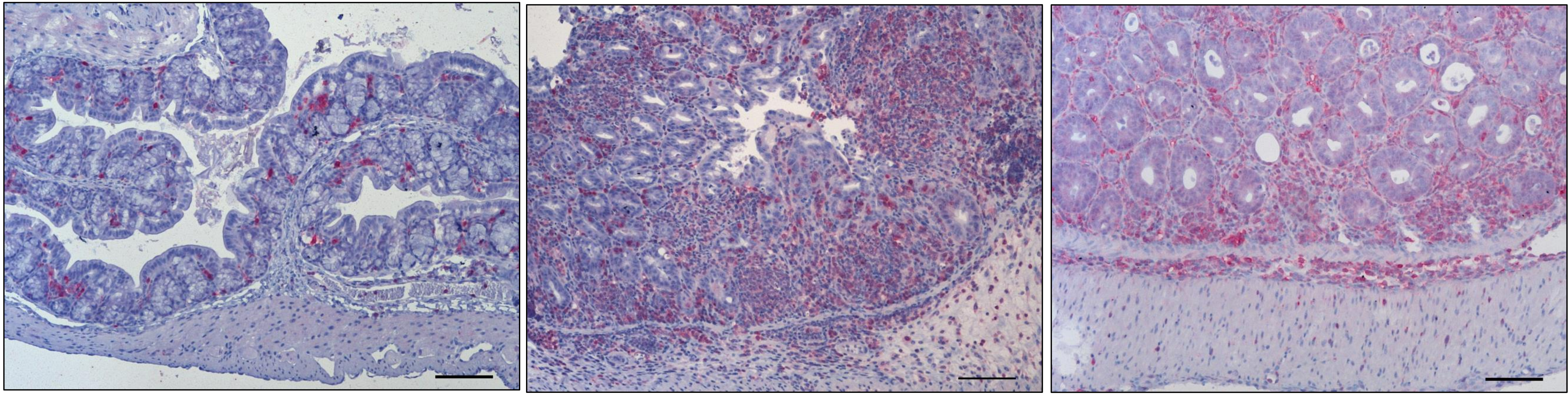

(100 x magnification, scale bar 100 µm)

E

Regulatory T Cells - COLON

None **Placebo** **Vitamin D**

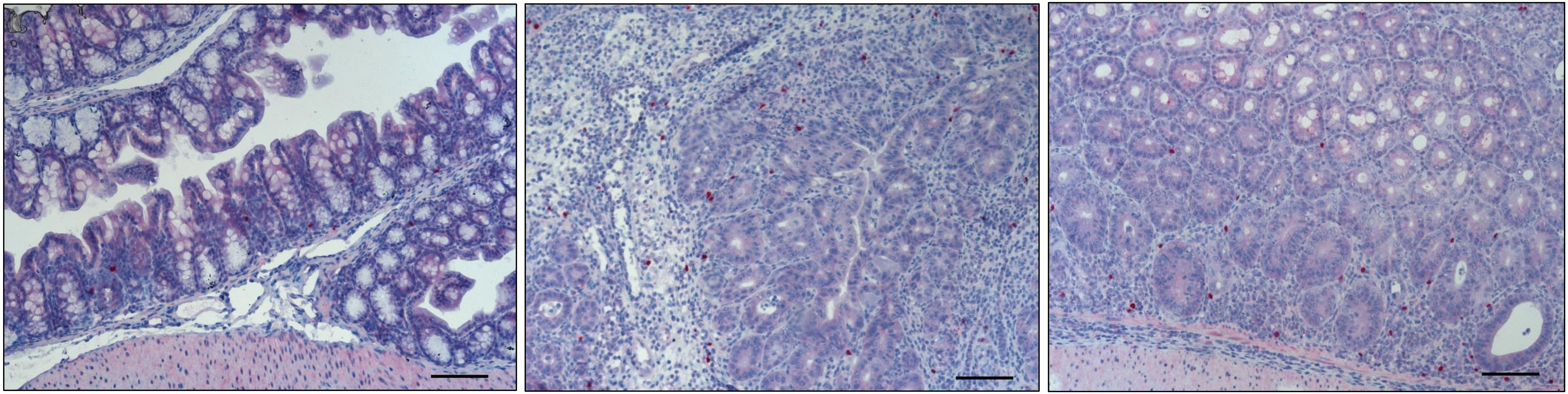

(100 x magnification, scale bar 100 μm)
